# Baicalin suppresses *Propionibacterium acnes*-induced skin inflammation by downregulating the NF-κB/MAPK signaling pathway and inhibiting activation of NLRP3 inflammasome

**DOI:** 10.1590/1414-431X20209949

**Published:** 2020-10-21

**Authors:** Fang Fang, Zeping Xie, Jingyu Quan, Xiaohan Wei, Linlin Wang, Liu Yang

**Affiliations:** School of Traditional Chinese Medicine, Southern Medical University, Guangzhou, China

**Keywords:** Acne, Baicalin, NF-κB, MAPK, NLRP3 inflammasome

## Abstract

Acne is a kind of common, chronic skin condition caused by the inflammation of the sebaceous glands in hair follicles. Recent studies have demonstrated that baicalin (BA) possesses potential anti-inflammatory properties. In this study, we evaluated the anti-inflammatory activity of BA *in vitro* and *in vivo*. Heat-killed *Propionibacterium acnes*-induced THP-1 cells and live *P. acnes*-injected male Sprague Dawley rats were used for establishing the acne model. The rate of ear swelling was calculated, and the severity was determined by hematoxylin and eosin staining. The production of cytokines [interleukin (IL)-1β, IL-6, IL-8, and tumor necrosis factor (TNF-α)] in the cell supernatant and ear tissue homogenates was measured by ELISA. Protein levels of JNK, ERK, P38, IκBα, P65, Nod-like receptor pyrin domain-containing 3 (NLRP3), pro-caspase-1, and IL-1β in THP-1 cells and ear tissues were detected by western blotting. NLRP3 and IL-1β were detected by immunohistochemistry, and the NLRP3, IL-1β and pro-caspase-1 mRNAs were detected by quantitative real-time polymerase chain reaction (qRT-PCR). The results showed that BA decreased the expression of pro-inflammatory cytokines *in vitro* and *in vivo*. Moreover, BA down-regulated the phosphorylation of JNK, ERK1/2, and κBα and inhibited the nuclear translocation of p65. Furthermore, BA inhibited the activation of NLRP3 inflammasome, at both the gene and protein levels. Taken together, the results demonstrated that BA might exert its anti-inflammatory activity by inhibiting NF-κB/MAPK signaling pathways and consequently suppressing the activation of the NLRP3 inflammasome both *in vivo* and *in vitro*.

## Introduction

Acne is a chronic skin condition caused by the inflammation of the sebaceous glands in hair follicles, and mainly occurs on the face, chest, and back, where the concentration of pilosebaceous glands is the highest (1). It clinically manifests as papules, pustules, nodules, and other pleomorphic skin lesions, and is often accompanied by the spillage of sebum. Acne vulgaris is a kind of common skin disease that mostly occurs in adolescents and young adults. In 2015, acne impacted more than 630 million people, and was the eighth most common disease worldwide (2). Nearly 1 billion dollars are annually spent for the treatment of acne in America alone, and more than $100 million are spent on over-the-counter medications for acne (2). Additionally, acne can cause emotional and psychological disturbances, resulting in complex feelings such as anxiety, inferiority, depression, social phobia, or even the intention of suicide (3). Currently, numerous studies have demonstrated that acne can result from several factors, including the excessive keratinization of hair follicles, excessive sebaceous secretion, colonization by microorganisms especially *P. acnes*, and disorders of inflammation and the immune response (4).

Although the definitive pathogenesis of acne is unclear, the overgrowth of *P. acnes* is considered to be a crucial factor in the formation of acne vulgaris (4). *P. acnes* is a common skin microorganism that predominantly resides in the pilosebaceous follicles of the human skin (4). It is recognized by toll-like receptor (TLR) 2 and TLR4, and activates inflammatory factors via the activation of MAPK kinases (MAPKs) and nuclear factor kappa-B (NF-κB) (5,6). Monocytes are important immune cells in the human body that provide the first line of defense against pathogens. Monocytes migrate from the blood stream to the inflamed skin of the acne in response to specific cytokines/chemokines, for clearing the pathogens. Monocytes and macrophages can also produce large amounts of proinflammatory cytokines, including interleukin (IL)-8, IL-1β, and tumor necrosis factor (TNF)-α. IL-8, a CXC chemokine, is a strong proinflammatory chemotactic factor for lymphocytes, basophils, and neutrophils. It is increased in keratinocytes by *P. acnes* stimulation (7). IL-1β is a crucial inflammatory factor, which is related to the activation of systemic and local responses to injury and infection. Acne lesions have high levels of IL-1β, which is thought to be a crucial driver of the inflammatory response in acne vulgaris, and is initiated by the activation of the NLRP3 inflammasome (8). The NLRP3 inflammasome comprises the NLRP3 protein, the ASC adaptor, and pro-caspase-1 (9). Owing to the key role of the NLRP3 inflammasome in the induction of inflammation in acne lesions, it is suggested that its suppression can be critical in the treatment of acne. The nuclear translocation and activation of NF-κB induces a marked increase in the expression of inflammatory factors, including IL-1β, IL-6, IL-8, and TNF-α (10). Studies have also demonstrated that NF-κB plays a key role during the activation of the NLRP3 inflammasome (11).

Presently, treatments for acne include topical therapies such as erythromycin, clindamycin, benzoyl peroxide, and retinoids, and systemic treatments such as oral antibiotics, oral retinoids, and hormonal therapy. However, these treatments often lead to irritant dermatitis (12,13), gastrointestinal disturbances (14), and bacterial resistance (15), among others. The primary disadvantage of the current therapies is antibiotic abuse, which can result in antibiotic resistance (16). The increasing resistance of *P. acnes* to topical macrolides has been reported in several countries (16). Although these therapies are still commonly used for the treatment of acne, other safer and more effective products are necessary. Recently, there has been an increasing number of studies on the use of other natural products for the treatment of acne.

Baicalin (BA) is a lipophilic flavonoid glycoside (17) isolated from *Radix Scutellariae*. It has been reported that BA possesses strong biological activities including anti-inflammatory (18), anti-oxidative (19), cardioprotective (20), anti-cancer (21), and anti-viral properties (22). So far, BA has not been found to have significant side effects. It has been reported that BA suppresses inflammation by suppressing the activation of the NLRP3 inflammasome and it alleviates age-related macular degeneration via miR-223/NLRP3-regulated pyroptosis (23). Additionally, BA mitigates cognitive impairment and protects neurons from microglia-mediated neuroinflammation by suppressing the activation of the NLRP3 inflammasome and the TLR4/NF-κB signaling pathway (24). However, the role and mechanism of BA in *P. acnes*-induced inflammation in acne has not been well explained. In the present study, the therapeutic effect and the underlying mechanism of BA on the treatment of *P. acnes*-induced acne were investigated.

## Material and Methods

### Reagents

BA (purity ≥95%) was purchased from Chengdu Mansite Ansite Biotechnology (China). BA was dissolved in DMSO and stored at −20°C. RPMI-1640 medium, fetal bovine serum (FBS), and penicillin/streptomycin were purchased from GIBCO/BRL Life Technologies (USA). Antibodies against ERK, phospho-ERK, p38, phospho-p38, JNK, phospho-JNK, phospho-IκB-α, p65, phospho-p65, caspase-1 p20, pro-caspase-1, IL-1β, and NLRP3 were purchased from Cell Signaling Technology (USA). GAPDH and anti-rabbit secondary antibodies were purchased from Abcam (USA). ELISA kits for IL-1β, IL-6, IL-8, and TNF-α were purchased from Dakewei (China). The kits for total RNA extraction and reverse transcription were purchased from Takara (Japan). The ECL kit was purchased from Affinity Biosciences (China). The kits for protein extraction and quantification were purchased from Keygen Biotech (China).

### *P. acnes* culture

*P. acnes* (ATCC, 6919, China) was obtained from the Guangdong Microbial Culture Center and grown under anaerobic conditions in 15 mL of brain heart infusion (China) at 37°C. The suspension of *P. acnes* was incubated at 80°C for 30 min for facilitating the heat-killing reaction. The suspension of heat-killed *P. acnes* was stored at 4°C until further analysis. Live *P. acnes* suspensions were preserved in glycerin at -80°C.

### Cell culture

Human monocyte THP-1 cells were maintained in RPMI-1640 supplemented with 10% FBS and 1% penicillin. The cells were cultured in an atmosphere of 5% CO_2_ at 37°C.

### Cell viability assay

Cell viability was determined by the CCK-8 assay. The cells were adjusted to a density of approximately 2×10^5^ cells/well in 96-well plates and treated with BA at various concentrations of 37.5, 75, 150, 300, and 600 μM for 24 h. Then, 10 μL of the CCK-8 reductant was added per well and allowed to incubate for a further 3 h at 37°C. The absorbance was measured spectrophotometrically at 450 nm on a microplate reader (Thermo Fisher Scientific, USA).

### *In vitro* acne model

The conditions for the generation of the acne-related inflammation model were the ratio of heat-killed *P. acnes* to the number of THP-1 cells of 100:1, and the incubation time was 12 h.

### Animals

Male Sprague Dawley rats (180-220g) were purchased from the Center of Experimental Animals of Southern Medical University (China). The rats were housed in climate-controlled quarters (22-26°C at 40-70% humidity) with a 12 h light/dark cycle and free access to food and water. All the experiments were performed in accordance with the guide for the care and use of laboratory animals by the National Institutes of Health, and were approved by the Ethical Committee for the Experimental Use of Animals at Southern Medical University (approval No. L2019111) (2015-002).

### Grouping and modeling

Forty-eight Sprague Dawley rats were randomly divided into the following six groups (n=8/group): control group (control), model group (*P. acnes*), treatment group that received a high dose of BA (H-BA), treatment group that received a medium dose of BA (M-BA), treatment group that received low dose of BA (L-BA), and doxycycline (DO) group. The outer contour of the right ear was used as the modeling site. PBS (40 μL) was intradermally injected into the right ear of the rats of the control group. PBS (40 μL) containing 5.0×10^8^ CFU of live *P. acnes* was intradermally injected into the right ear of the rats in the model group. The control group received normal saline by gavage at a dose of 1 mL/day. The BA groups were administered BA by gavage at low dose (25 mg/kg per day) (L-BA group), medium dose (50 mg/kg per day) (M-DA group), or high dose (100 mg/kg per day) (H-BA group). The positive control group was treated with DO tablets at a dose of 12.6 mg/kg/day. The DO tablets were dissolved by normal saline and administered to the rats by gavage. The dose of BA was selected according to the clinical dose, and converted into an equivalent dosage for rats. The rats received the intragastric treatment on the third day after the injection of *P. acnes*. After 7 days of treatment, the rats were anesthetized with 5% (w/v) pentobarbital sodium and euthanized. The thickness of the ears was measured using micro-calipers, after which the ears were excised and placed in liquid nitrogen for grinding. After homogenization, the related indicators were detected. The remaining ear samples were embedded in paraffin and stained with H&E for histological examination.

### ELISA

THP-1 cells (1.0×10^5^ cells/well) were pre-treated with BA at various concentrations of BA (75, 150, and 300 μM) for 24 h, followed by stimulation with heat-killed *P. acnes* for 12 h in 24-well plates. The levels of IL-6, IL-8, IL-1β, and TNF-α of cell supernatants were determined using ELISA kits according to the manufacturer's instructions. The absorbance of the ELISA plate was measured at 405 nm by a microplate reader. After the right ear tissue was homogenized, the levels of IL-6, IL-8, IL-1β, and TNF-α of the homogenized tissue were detected by ELISA.

### Western blot analysis

THP-1 cells (4.0×10^5^ cells/well) were pre-treated with BA at concentrations of 75, 150, and 300 μM for 24 h, and subsequently stimulated with heat-killed *P. acnes* for 12 h in 6-well plates. There were 5 groups: blank control group, model group, and 3 BA groups. In order to extract the total protein from the cultured cells, the harvested cells were rinsed with PBS and lysed using lysis buffer (Keygen Biotech, China) for 15 min on ice. The samples were centrifuged at 15,000 *g* at 4°C for 20 min. The supernatants were subsequently harvested and stored at −20°C. The nuclear extract was prepared according to the protocol described in the nuclear and cytoplasmic protein extraction kit. The concentrations of cell lysates and nuclear lysates were quantified using the BCA assay kit (Keygen Biotech). The protein was then mixed with 5× sample buffer and boiled at 95°C for 5 min. An equal amount of the protein samples was separated by 10% SDS-polyacrylamide gel electrophoresis (PAGE) and transferred to polyvinylidene fluoride (PVDF) membranes (Bio-Rad, USA). The membranes were blocked with 5% skim milk in TBST for 1 h at room temperature, and incubated overnight with primary antibodies (1:1000) at 4°C. After washing thrice with TBST, the membranes were incubated with the secondary antibodies (1:1000) for 1 h at room temperature. The protein-antibody conjugates were visualized using the ECL kit by the FluorChem E™ system (ProteinSimple, USA). The expression of the proteins in the auricle tissues was detected by western blotting. The right ear of the rat was excised on the 8th day after treatment, and subsequently homogenized in the protein lysate with a hand tissue grinder. The rest of the experimental steps were as described before.

### qRT- PCR

THP-1 cells (4.0×10^5^ cells/well) were pre-treated with BA at concentrations of 75, 150, and 300 μM for 24 h, and subsequently stimulated with heat-killed *P. acnes* for 12 h in 6-well plates. There were 5 groups: blank control group, model group, and 3 BA groups. The total RNA was isolated from THP-1 cells using TRIzol reagent (Sigma, USA). The purity of the RNA was detected by spectrophotometry. The total RNA was used as the template for cDNA synthesis using a PrimeScript RT Master Mix (Takara, Japan), according to the manufacturer's instructions. Then qRT-PCR was performed with SYBR‐green (Takara) in a Roche Light Cycler^®^ 96 System (Roche, Switzerland). Each sample was tested three times. The relative gene expression profiles were determined by normalizing the expression to that of the reference gene (GAPD), using the 2^−ΔΔCt^ method. The expression of the transcription factors in the ear tissues was detected by qRT-PCR. The right ear of the rat was excised on the 8th day after treatment, and subsequently homogenized in TRIzol Reagent with a hand tissue grinder. The remaining experimental steps were the same as described before. The primer sequences used for qRT-PCR are shown in Table 1.

**Table 1:** Primer sequences used in the study.

Primer	Sequence
Human IL-1β	
Forward	GCCAGTGAAATGATGGCTTATT
Reverse	AGGAGCACTTCATCTGTTTAGG
Human NLRP3	
Forward	GCAGCGATCAACAGGCGAGA
Reverse	TCCCAGCAAACCTATCCACTCCTC
Human pro-caspase-1	
Forward	GAAGAAACACTCTGAGCAAGTC
Reverse	GATGATGATCACCTTCGGTTTG
Rat IL-1β	
Forward	AATCTCACAGCAGCATCTCGACAAG
Reverse	TCCACGGGCAAGACATAGGTAGC
Rat NLRP3	
Forward	CTGCTGTGCGTGGGACTGAAG
Reverse	AGAACCAATGCGAGATCCTGACAAC
Rat pro-caspase-1	
Forward	GCACAAGACTTCTGACAGTACCTTCC
Reverse	GCTTGGGCACTTCAATGTGTTCATC

### Histopathological and immunohistochemical examination of ear tissues

The tissues of the right ears were fixed in 4% (w/v) paraformaldehyde for 24 h. The samples were dehydrated in graded ethanol and embedded in paraffin. Some of the 5 μm-thick sections were stained with H&E and observed by light microscopy (Olympus IX53, Japan). The tissues of the other ears were incubated in citrate antigen retrieval solution (Boster) for 10 min at 95°C and cooled down to room temperature. The sections were washed and incubated in 0.3% H_2_O_2_ for 10 min for quenching the activity of endogenous peroxidases prior to blocking with 10% BSA in PBS at room temperature for 2 h. The slides were then incubated overnight with NLRP3 and IL-1β antibodies (1:800) at 4°C and subsequently incubated with the secondary antibody conjugated with horseradish peroxidase (1:1000) for 45 min at 37°C. Specific labeling was performed with the DAB substrate kit, and the specimens were counterstained with hematoxylin prior to observation by light microscopy (Olympus IX53).

### Statistical analyses

The data are reported as means±SE from at least three independent experiments. In order to compare the mean values among the multiple groups, one-way analysis of variance (ANOVA) was performed, followed by least significant difference *post hoc* tests or Tukey test. All the analyses were performed using the statistical software GraphPad Prism version 5.0 (USA). The data were considered to be statistically significant when P<0.05.

## Results

### BA suppressed the production of pro-inflammatory cytokines induced by *P. acnes* in THP-1 cells

**Figure 1:**
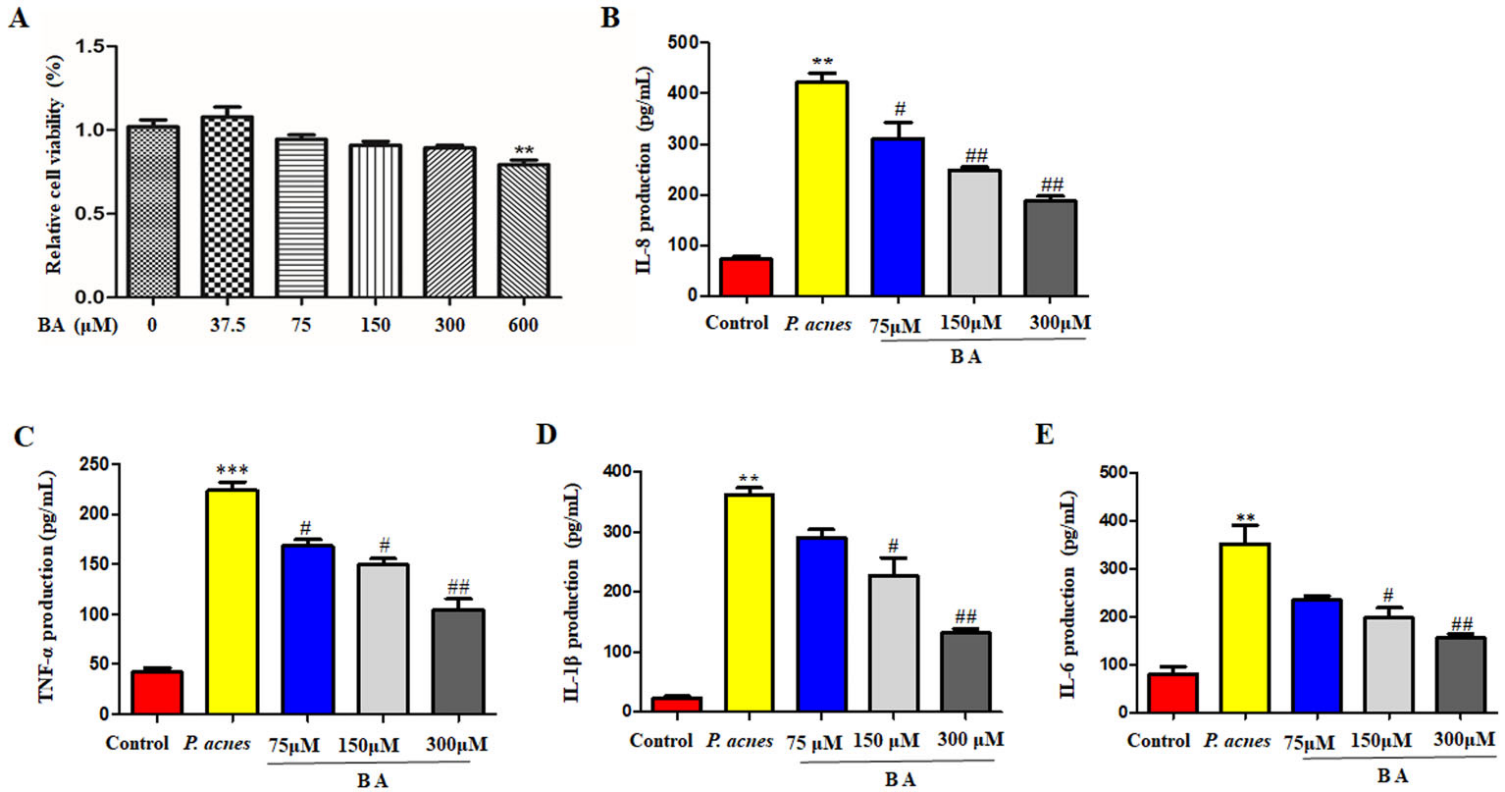
Effects of baicalin (BA) on *P. acnes-*induced cytokine production in THP-1 cells. **A**, Survival rate of THP-1 cells following treatment with BA at concentrations of 37.5, 75, 150, 300, and 600 μM for 24 h was evaluated using the CCK-8 kit. **B**-**E**, Levels of interleukin (IL)-8, tumor necrosis factor (TNF)-α, IL-1β, and IL-6, in *P. acnes*-stimulated THP-1 cells quantified by ELISA. The data are reported as means±SE from at least three independent experiments. **P<0.01, ***P<0.001 *vs* control; ^#^P<0.05, ^##^P<0.01 *vs P. acnes* alone (ANOVA).

In order to study the potential effects of BA on *P. acnes*-induced inflammation, we first determined the non-cytotoxic concentration of BA in THP-1 cells. The cell counting kit (CCK)-8 assay was used to investigate cytotoxicity. The result showed that BA at concentrations up to 300 μM had no obvious cytotoxicity after 24 h (Figure 1A). Next, the effect of BA on heat-killed *P. acnes*-induced pro-inflammatory cytokine production of THP-1 cells was determined. BA suppressed the secretion of IL-1β, IL-6, IL-8, and TNF-α in THP-1 cells (Figure 1B-E).

### Effect of BA on MAPK and NF-κB signaling pathways in THP-1 cells

**Figure 2:**
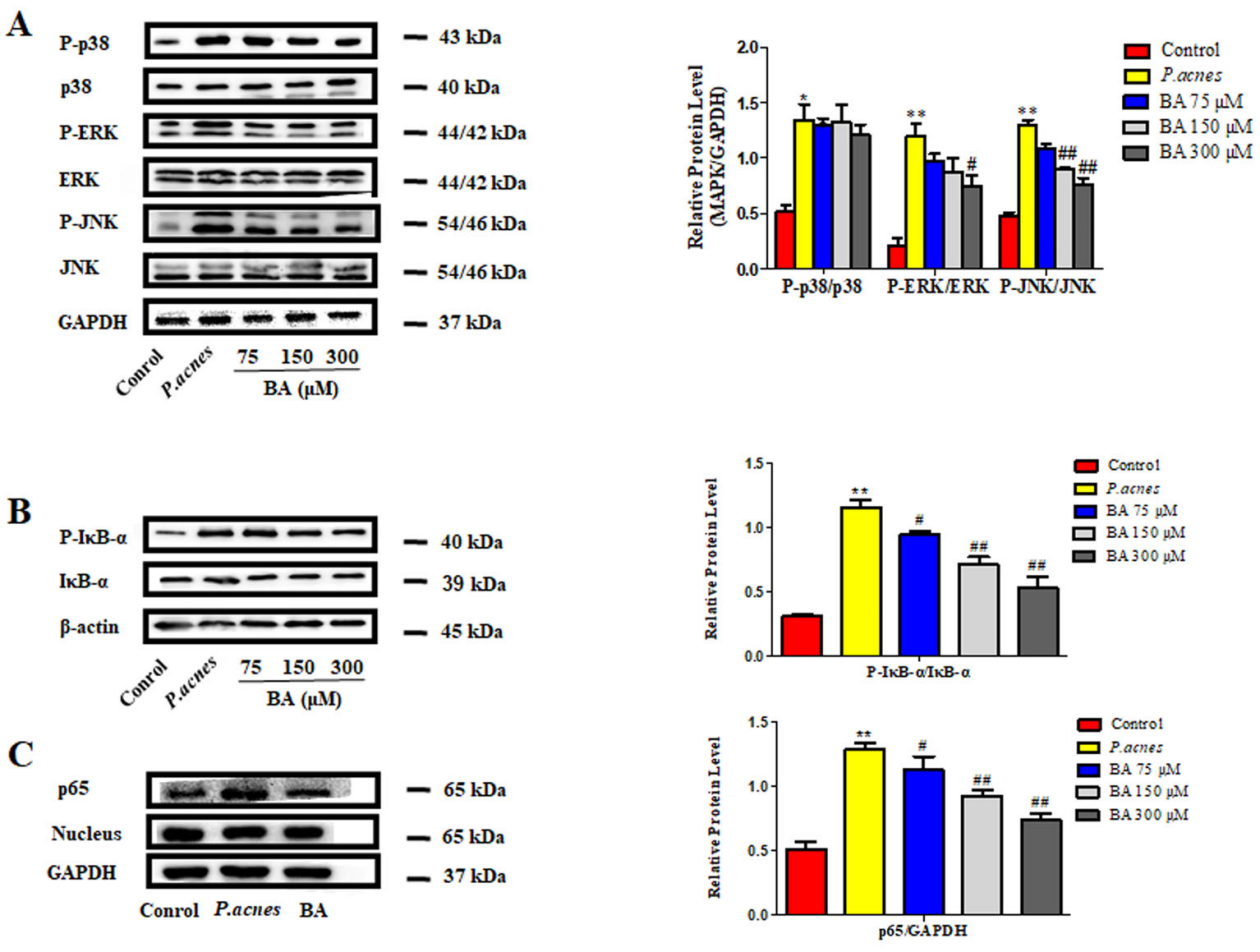
Effects of baicalin (BA) on the MAPK and NF-κB signaling pathways in *P. acnes*-stimulated THP-1 cells. THP-1 cells were pre-treated with different concentrations of BA (75, 150, 300 μM) for 24 h, and then stimulated with heat-killed *P. acnes* for a further 12 h. Total cell lysates and nuclear lysates were separated by gel electrophoresis and specific proteins were measured by western blotting. **A-C**, Detection of phosphorylated ERK, JNK, p38, IκBα, and the NF-κB p65 nuclear translocation. The data are reported as means±SE from at least three independent experiments. *P<0.05, **P<0.01 *vs* control; ^#^P<0.05, ^##^P<0.01 *vs P. acnes* alone (ANOVA).

As depicted in Figure 2A, the levels of phosphorylated ERK, JNK, and p38 increased after stimulation with *P. acnes*, and the phosphorylation of ERK1/2 and JNK was reduced in a dose-dependent manner in the BA-treated cells. However, there were no significant changes in the expression of phosphorylated p38. We also examined the effect of BA on the activation of the transcription factor NF-κB, which plays a significant role in inducing the secretion of inflammatory factors. We monitored the phosphorylation of IκBα and the subsequent nuclear translocation of the p65 subunit of NF-κB. BA effectively suppressed the phosphorylation of IκBα in *P. acnes*-stimulated THP-1 cells (Figure 2B) and inhibited the nuclear translocation of the p65 subunit of NF-κB (Figure 2C). These results indicated that BA inhibits both the MAPK and NF-κB signaling pathways in *P. acnes*-stimulated THP cells.

### BA inhibited the activation of the NLRP3 inflammasome *in vitro*

**Figure 3:**
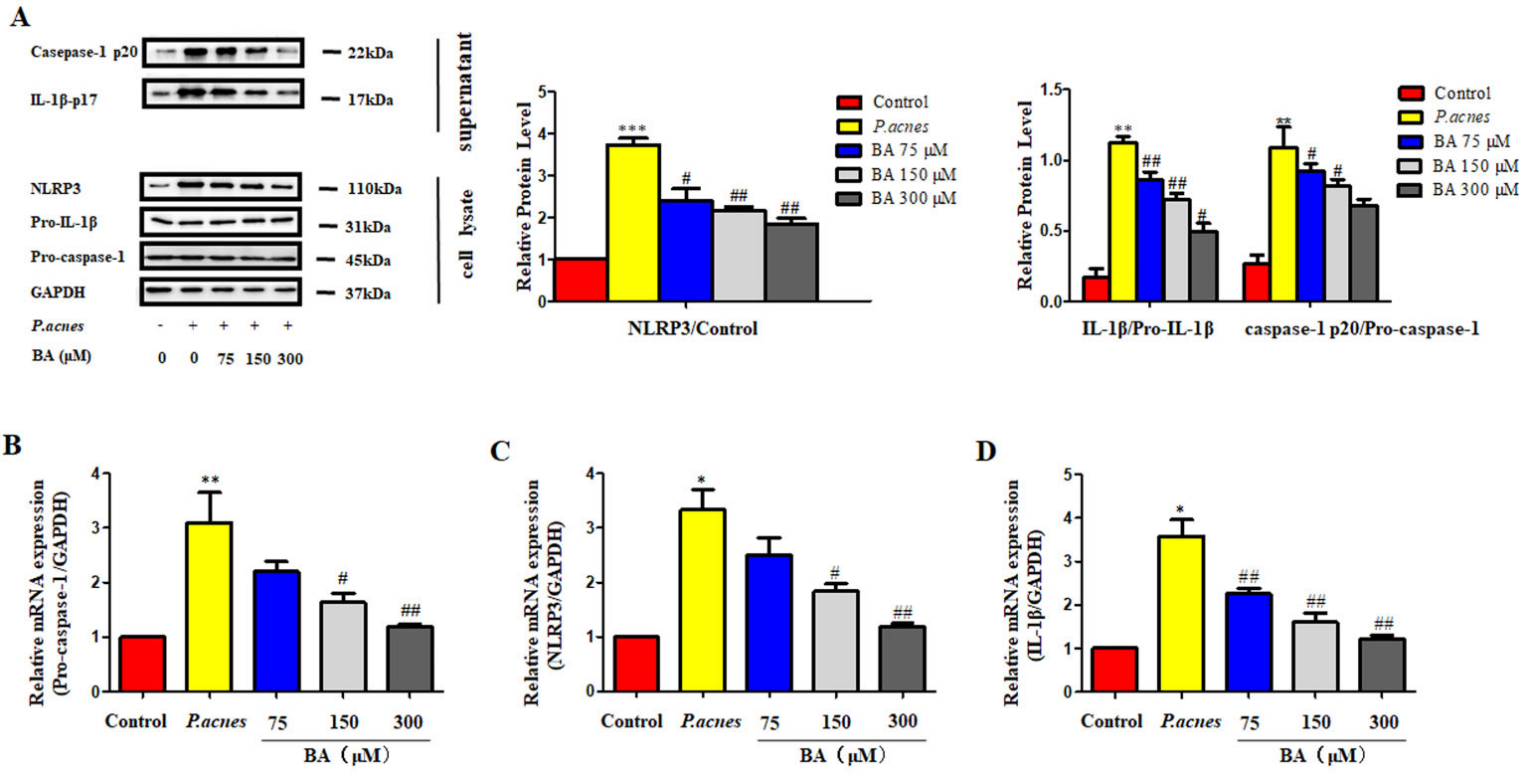
Effect of baicalin (BA) on the expression of interleukin (IL)-1β and the NLRP3 inflammasome in *P. acnes-*induced THP-1 cells. The THP-1 cells were pre-treated with BA at concentrations of 75, 150, and 300 μM for 24 h, and subsequently stimulated with heat-killed *P. acnes* for 12 h in 6-well plates. **A**, The cell supernatants were harvested for the detection of IL-1β and caspase-1 p20. Western blotting was performed for assessing the expression of NLRP3 and pro-caspase-1. **B**-**D**, qRT-PCR for quantifying the mRNA levels of pro-caspase-1, NLRP3, and IL-1β. The data are reported as means±SE from at least three independent experiments. *P<0.05, **P<0.01, ***P<0.001 *vs* control; ^#^P<0.05, ^##^P<0.01 *vs P. acnes* alone (ANOVA).

The NLRP3 inflammasome regulates inflammation by inducing the production and maturation of IL-1β (26). The results of western blotting revealed that BA inhibited the production of IL-1β p17 and caspase-1 p20, and inhibited the expression of NLRP3 (Figure 3A). We also monitored the effect of BA on the NLRP3 inflammasome following stimulation by *P. acnes* using qRT-PCR. The levels of IL-1β, pro-caspase-1, and NLRP3 mRNA transcripts induced by *P. acnes* were significantly suppressed by BA (Figure 3B-D). Together, these results suggested that treatment with BA regulated the NLRP3-dependent pro-inflammatory processes that were induced by *P. acnes* in THP-1 cells.

### BA ameliorated the *P. acnes*-induced inflammation *in vivo*

**Figure 4:**
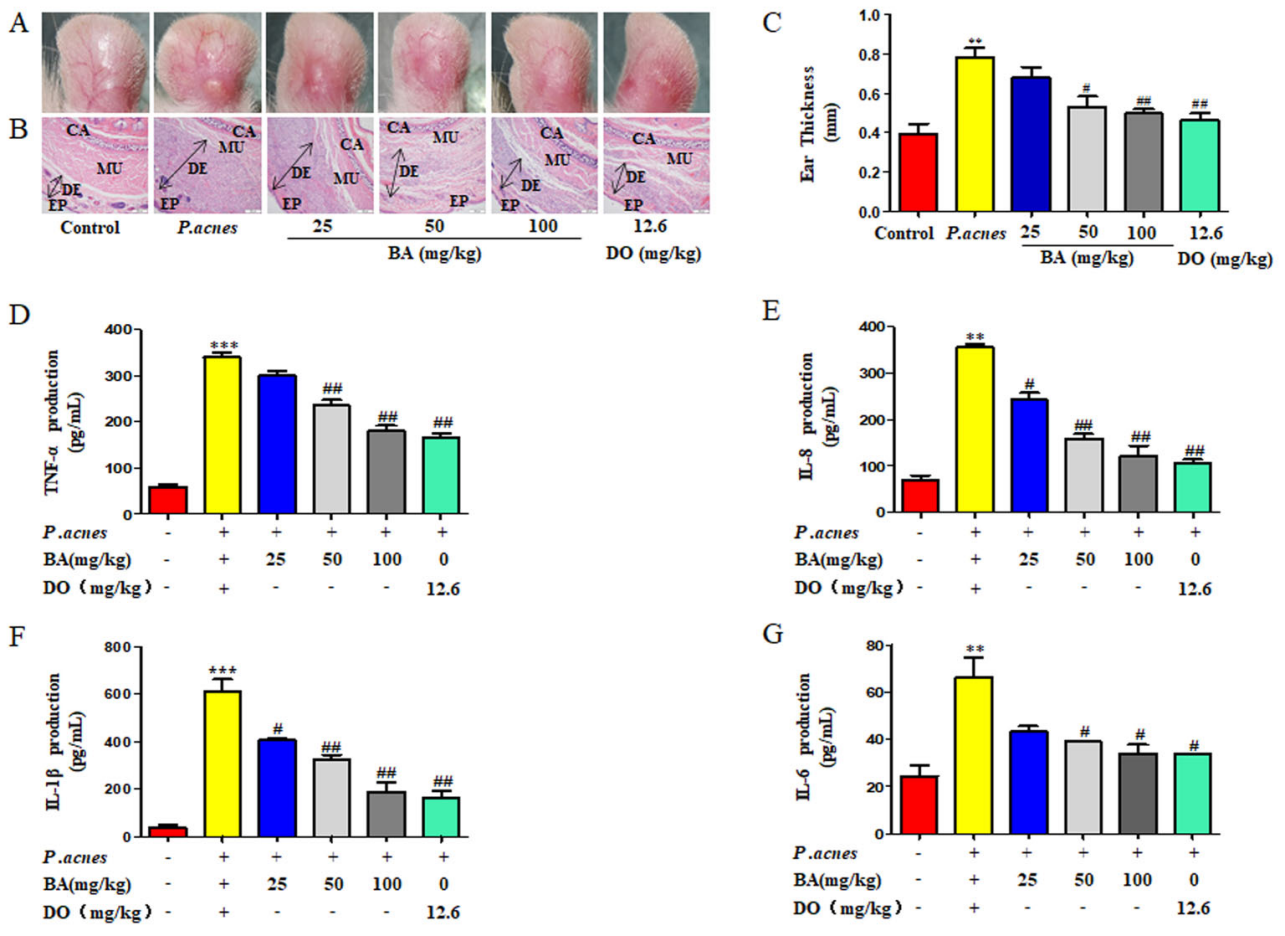
Baicalin (BA) ameliorated the *P. acnes*-induced skin inflammation in the ears *in vivo.* The formalin-fixed ear sections were subjected to H&E staining (scale bar=200 μm). **A** and **B**, The images are representative of eight independent samples, and the length of the double headed arrows represents the thickness of the dermis. **C**, The thickness of the ears was measured using a micro-caliper prior to euthanasia. The production of tumor necrosis factor (TNF)-α (**D**), IL-8 (**E**), IL-1β (**F**), and IL-6 (**G**) in the ear tissue homogenates was quantified by ELISA. CA: ear hyaline cartilage; EP: epidermis; DE: dermis; MU: muscle; DO: doxycycline. The data are reported as means±SE from at least three independent experiments. **P<0.01, ***P<0.001 *vs* control; ^#^P<0.05, ^##^P<0.01 *vs P. acnes* alone (ANOVA).

Histological examination revealed that the inoculation of *P. acnes* induced swelling and increased the infiltration of inflammatory cells into the dermis (Figure 4A), but treatment with BA significantly attenuated these changes (Figure 4B) and reduced ear thickness (Figure 4C). The *P. acnes-*induced production of IL-1β, IL-6, IL-8, and TNF-α pro-inflammatory cytokines decreased consistently and proportionally following BA treatment (Figure 4D-G). These results indicated that BA ameliorated the *P. acnes*-induced inflammation of the ear skin of rats.

### Effects of BA on the MAPK and NF-κB signaling pathways

**Figure 5:**
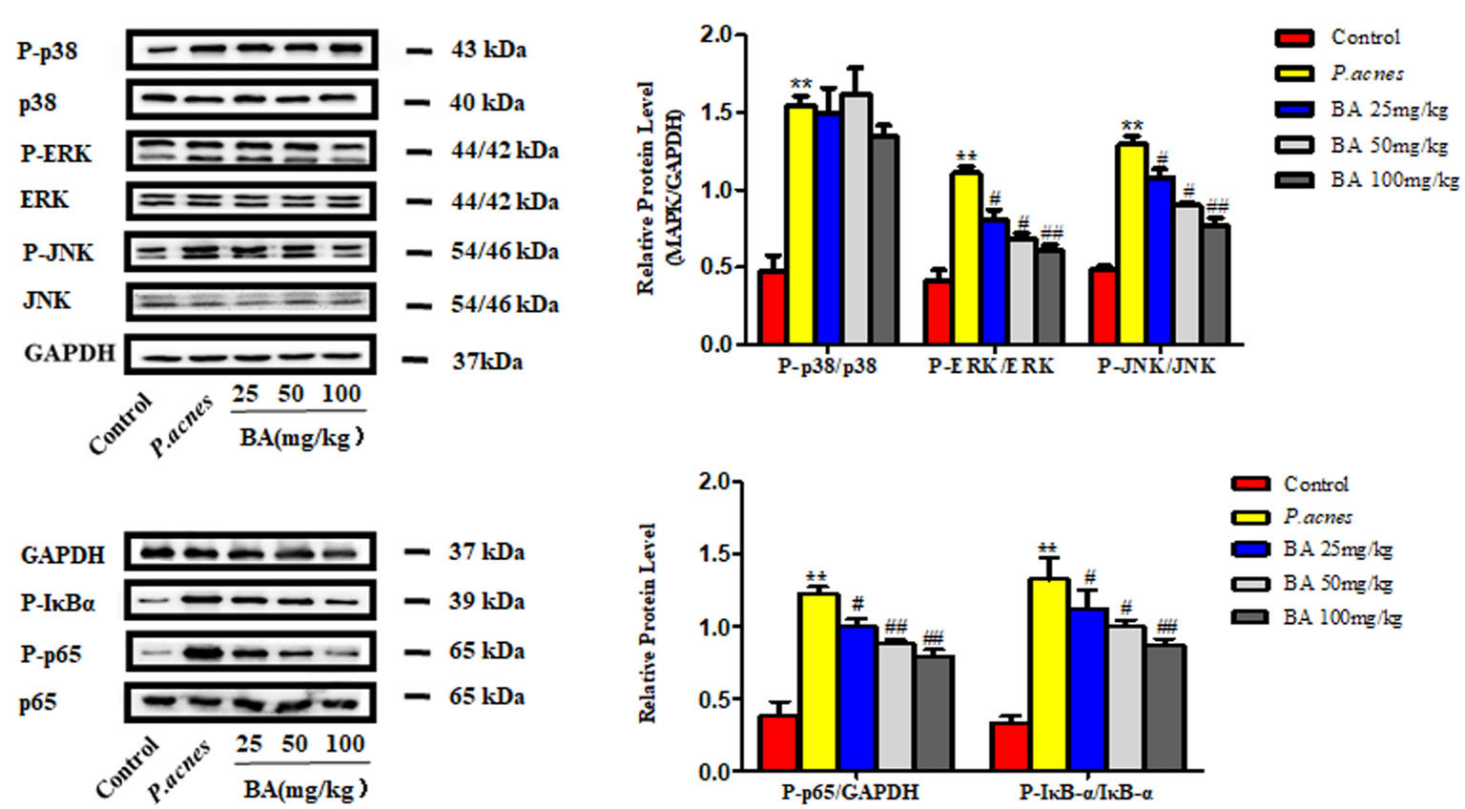
The effects of baicalin (BA) on the MAPK and NF-κB signaling pathways were assessed *in vivo*. The protein levels of ERK, p38, JNK, IκBα, and P-p65 in the ear tissues of the rats were assessed for each group. The levels of P-ERK, P-p38, P-JNK, and P-p65 were normalized to the levels of ERK, p38, JNK, and p65 proteins, respectively. GAPDH was used as the internal loading control for p-IκBα. The data are reported as means±SE from at least three independent experiments. **P<0.01 *vs* control; ^#^P<0.05, ^##^P<0.01 *vs P. acnes* alone (ANOVA).

As shown in Figure 5, the levels of phosphorylated ERK, JNK, and p38 increased following stimulation with *P. acnes*, and the phosphorylation of ERK and JNK decreased in a dose-dependent manner following treatment with BA. However, there were no significant changes in the expression of phosphorylated p38. The results further demonstrated that BA abolished the *P. acnes*-induced increase in P-IκBα and P-p65 in a dose-dependent manner. The results of the *in vivo* experiments with consistent with those of the *in vitro* experiments.

### BA suppressed the activation of the NLRP3 inflammasome *in vivo*

**Figure 6:**
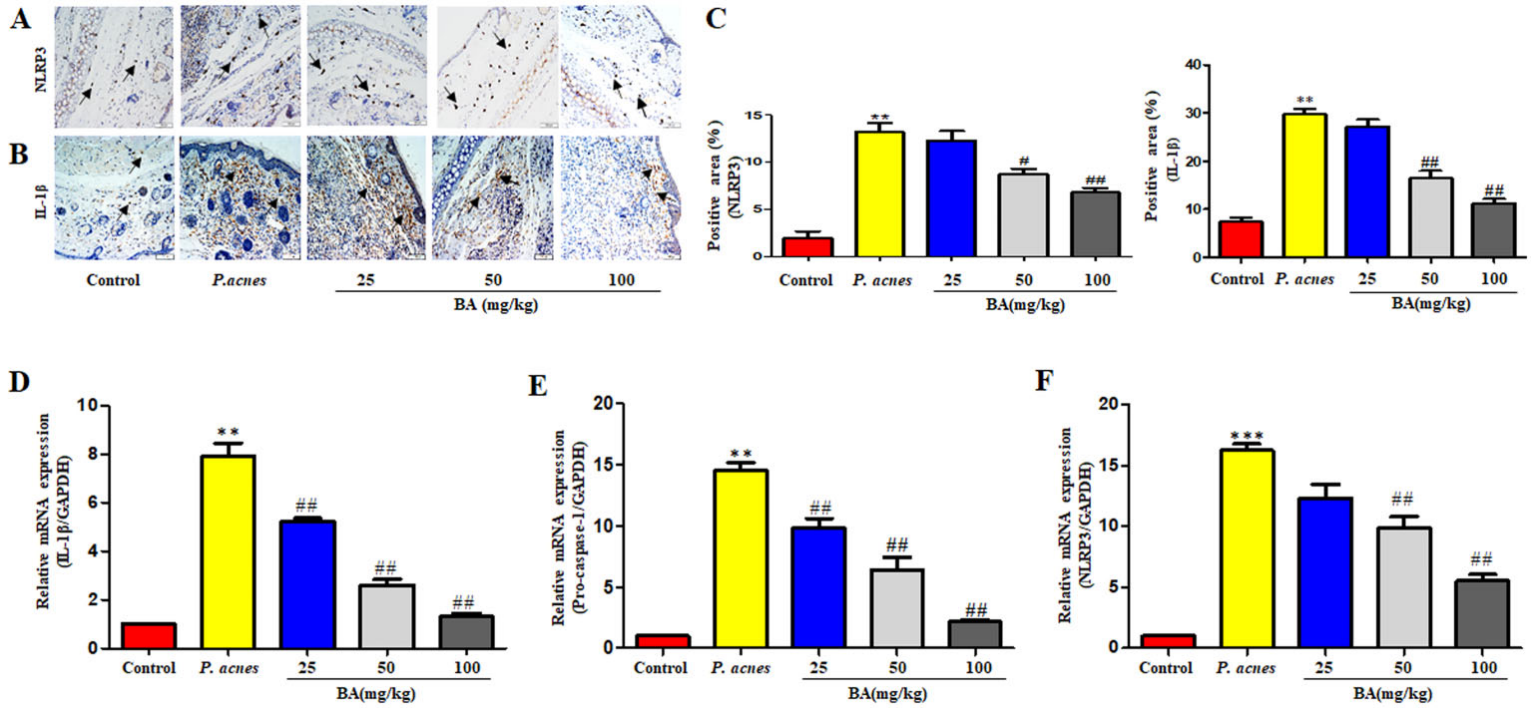
Effect of baicalin (BA) on *P. acnes*-induced activation of the NLRP3 inflammasome in the ears. **A** and **B**, Representative images of the immunohistochemical staining of the ear tissue sections for studying the expression of NLRP3 and interleukin (IL)-1β (scale bar=100 μm). **C**, Quantification of the results shown in panels A and B. **D-F**, Quantification of IL-1β, pro-caspase-1, and NLRP3 mRNA transcription levels by qRT-PCR. The data are reported as means±SE from at least three independent experiments. **P<0.01, ***P<0.001 vs control; ^#^P<0.05, ^##^P<0.01 *vs P. acnes* alone (ANOVA).

We further investigated the effect of BA on the activation of the NLRP3 inflammasome in *P. acnes*-induced ear inflammation in rats. Immunohistochemical analysis of the ear tissue sections revealed that the expression of NLRP3 and IL-1β increased following stimulation with *P. acnes* (Figure 6A and B). BA significantly reduced the expression of NLRP3 and IL-1β (Figure 6C). Similar results were also observed by qRT-PCR analysis (Figure 6D-F). The expression of NLRP3 and IL-1β mRNAs in the *P. acnes*-inoculated group was significantly higher than that of the control group, and this increase was significantly suppressed following the administration of BA at concentrations of 25, 50, and 100 mg/kg, twice a day. These results indicated that the anti-inflammatory properties of BA are partly mediated by the suppression of the activation of the NLRP3 inflammasome.

## Discussion

The role of *P. acnes* in the inflammatory response of acne vulgaris has been recognized for over a century. Although the exact pathogenesis of acne vulgaris is yet to be understood, it has been verified that the colonization and proliferation of *P. acnes* is crucial to the development of inflammation (25). The peptidoglycan cell wall of *P. acnes* initiates the release of cytokines such as IL-1β, IL-8, and TNF-α by monocytes, which causes granulomatous inflammatory responses in the skin (26,27). The results of our study demonstrated that BA possesses significant anti-inflammatory properties in *P. acnes*-induced inflammation, as revealed by *in vivo* and *in vitro* experiments.

Heat-killed *P. acne*-induced THP-1 cells were used as the *in vitro* model. We first demonstrated that the production of inflammatory factors was increased after stimulating THP-1 cells with heat-killed *P. acnes*. We also confirmed that BA reduced the production of inflammatory factors in a dose-dependent manner. Under inflammatory stimulation, NF-κB activation occurs through the phosphorylation of IκB-α and subsequently causes p65 nuclear translocation. Nuclear p65 activates the transcription of pro-inflammatory mediators (28,29). We demonstrated that BA effectively inhibited the *P. acnes*-induced phosphorylation of IκB-α and the nuclear translocation of the p65 subunit of NF-κB in THP-1cells, indicating that BA inhibited NF-κB signaling. Furthermore, MAPK is critical for the activation of NF-κB signaling and the binding of NF-κB to pro-inflammatory genes (30,31). NF-κB, an important downstream target of MAPK signaling, regulates various genes encoding the pro-inflammatory mediators of inflammatory and immune responses (32,33).

In this study, the phosphorylation of ERK1/2, JNK, and p38 significantly increased in THP-1 cells following stimulation by *P. acnes.* The results of this study demonstrated that BA significantly inhibited the phosphorylation of ERK and JNK, but not that of p38. Indeed, previous studies have suggested that ERK plays both pro- and anti-inflammatory roles depending on the inducers and cell types (34). Furthermore, it has been suggested that JNK is a potential target for the treatment of inflammatory diseases (35). The results of this study were in line with these reports. p38 MAPK is widely involved in intracellular signaling cascades that mediate inflammatory responses. These findings indicated that BA may exert its anti-inflammatory activity by inhibiting the NF-κB pathway and suppressing the activation of the JNK and ERK1/2 MAPK signaling pathways.

The maturation of IL-1β depends on the activation of pro-caspase-1, and the processing and release of IL-1β are closely related to NLRP3 in response to various pathogens. The NLRP3 inflammasomes comprise ASC, NLRP3, and pro-caspase-1. The activation of the NLRP3 inflammasome and the maturation of IL-1β involve a two-step mechanism. The peptidoglycan cell wall of *P. acnes* may play a key role in the first step of TLR signaling. TLR signaling induces the NF-κB-dependent expression of genes encoding NLRP3, pro-IL-1β, and pro-IL-18. It has been reported that NF-κB and MAPK signaling are critical for the activity of the NLRP3 inflammasome in numerous cell types, including mammary epithelial cells. Secondly, the assembly of the NLRP3 inflammasome leads to the activation of caspase-1, which converts pro-IL-1β and pro-IL-18 into their active forms; these eventually caused the death of the cells. In our study, BA suppressed both the NF-κB pathway and the activation of the ERK1/2 MAPK signaling pathways, and significantly inhibited the expression of caspase-1 p20, and NLRP3 protein. These results indicated that BA was capable of inhibiting the activation of inflammasomes. Therefore, the inhibition of both the activation of the NLRP3 inflammasome and the production of IL-1β are considered to be latent therapeutic targets of treating acnes.

The results of our study demonstrated that BA could potentially reduce and treat *P. acnes*-induced inflammation by suppressing the activation of the NLRP3 inflammasome via the NF-κB/MAPK pathway.
